# A Hypothetical Vascular Stent with Locally Enlarged Segment and the Hemodynamic Evaluation

**DOI:** 10.1155/2020/7041284

**Published:** 2020-02-25

**Authors:** Yudi Niu, Anqiang Sun, Zixuan Wang, Chenghong Yao, Juxingsi Song

**Affiliations:** ^1^Key Laboratory for Biomechanics and Mechanobiology of Ministry of Education, School of Biological Science and Medical Engineering, Beihang University, Beijing 100083, China; ^2^Beijing Advanced Innovation Center for Biomedical Engineering, Beihang University, Beijing 100083, China

## Abstract

Among the interventional stenting methods for treating coronary bifurcation lesions, the conventional treatments still have disadvantages, which include increased intervention difficulties or inadequate supply of blood flow to side branches and may alter the physiological function of downstream organs. Thus, the optimized design of stent geometry needs to be improved based on the specific shape of branches to minimize the complications of inadequate blood flow to the downstream organs and tissues. Our research used 3D modeling and fluid dynamics simulation to design and evaluate a new stent with locally enlarged segment by altering the proportion and length of enlarged surface area based on Bernoulli's equation. The aim is to increase the pressure and blood flow supply at side branches. According to series of blood flow simulations, the stent with 10% enlargement of surface area and length of 3 folders of stent diameter was assigned as the optimized design. The results revealed that by using this design, according to the simulation results, the average pressure on side branches increased at the rate of 43.6%, which would contribute to the adequate blood supply to the downstream organs. Besides, the average wall shear stress (WSS) at sidewalls increased at 9.2% while the average WSS on the host artery wall decreased at 14.1%. There is in the absent of noticeable rise in the total area of low WSS that blows the threshold of 0.5 Pa. Therefore, the present study provides a new method to optimize the hemodynamics features of stent for bifurcation arteries.

## 1. Introduction

Coronary bifurcation lesion mainly refers to the presence of larger than 50% of stenosis in the main vessel and branch vessel openings [1]. It accounts for about 15% to 20% of coronary interventional therapy [2] and have low surgical success rate [3], high risk of intraoperative occlusion during percutaneous coronary angioplasty [3, 4], as well as high rate of postoperative branch restenosis [5]. Due to its anatomical structure, there is no standard guideline for the treatment of branch lesions in clinical practice [6]. Coronary branch lesion itself is an independent risk factor for stent thrombosis after Percutaneous Coronary Intervention (PCI) [7] and has relatively low long-term intervention safety [6, 8].

Single-stent intervention is a simple treatment strategy that can significantly shorten the intervention time and complexity during clinical practice, but this oversimplified surgical approach which emphasizes the provisional stent strategy would lead to the potential decrease of blood supply [9] in side branches and finally induces occlusion, local myocardial necrosis [10], and increased perioperative myocardial infarction [11]. Thus, double-stent intervention is designed to cover the main vessels and side branches at the meantime to guarantee sufficient blood flow and avoid residual stenosis in the lateral branch. However, there are also some disadvantages for double-stent intervention strategy including incomplete coverage, vascular dissegment, high rate of thrombus and restenosis, etc. [12, 13].

Both the existing single-stent and double-stent intervention techniques have difficulties in avoiding shielding of the bifurcation vessels [14, 15], causing increased restenosis rate and insufficient blood supply to downstream organs, which may alter the corresponding physiological functions [14].

As a result, we proposed a novel design based on Bernoulli's equation [16] (equation (1)) to simultaneously ensure the adequate blood supply to bifurcation vessel and lower risk of restenosis rate with enlarged segment at branches.

(1) $$\begin{array}{c} \frac{v^{2}}{2}+gz+\frac{p}{\rho }=\text{constant,} \end{array}$$

where *g* is the gravitational acceleration, *v* is the fluid flow speed of a streamline point, *z* is the elevation of the point above a reference plane, *p* is the pressure at the targeted point, and *ρ* is the density of the fluid.

The above equation suggests that when the fluid velocity decreases, the fluid pressure may increase, which can improve the blood supply and avoid blockage in clinical treatment of atherosclerosis. Given consideration to that the energy difference caused by the changes of elevation in blood flow is minor and can be neglected [17, 18], it is obvious to draw the conclusion that the decrease in velocity of blood contributes to the rise of blood supply pressure and may compensate for the inadequate blood supply near branch lesions. The decrease of blood velocity may result from the enlargement of cross-segment area of vessel when the flow rate remains the same or appears in the flow stagnant zone [19] of disturbed laminar flow where Reynold's number exceeds 200 [20]. Our research is based on the theory that aims at lifting the blood supply pressure by designing enlarged segment in the vascular stent at side branches. It also provides reliable theoretical analysis and experimental data for clinical applications. At the same time, based on the single-stent intervention strategy, the operational difficulty of the existing technique would not increase the implantation difficulties. However, the potential decrease in wall shear stress should be monitored, which may augment the risk of disturbed hemodynamics pattern and atherosclerosis.

The goal of the present study is to investigate the hemodynamic improvement after the use of a stents with enlarged segment at the bifurcated vessel according to the ideal vascular models with variable controls (length and percentage of the enlarged area) through quantitative calculation of WSS and pressure to determine the optimized parameter of the vascular stent design. Finally, the ideal design has been selected based on the criteria of increased flow pressure (in the absence of abrupt decrease in wall shear stress as well) and adequate blood supply to the downstream organs.

## 2. Methods

Before the simulation of ideal vascular models, a series size of vascular stents without enlargement and with certain percentage of enlargement at 5%, 10%, 15%, 20%, 25%, and 30% (the clinical standard upper limit) were constructed according to the clinical data of PCI treatment [21]. With the length of the fixed expansion varied from (*L* (length) = *D* (main vessel diameter) to *L* = 3*D*), we explored the blood flow pressure distribution at the bifurcation vessel after stent implantation to determine the enhancement of blood flow supply. At the same time, the average WSS at the main and branch vessels were examined and calculated to monitor the potential increased risk of atherosclerosis due to the decrease of blood flow velocity. Consequently, an optimized design is defined based on the selecting criteria of maximum increase in blood pressure and minimum decrease in WSS.

### 2.1. Modeling in SolidWorks

**Figure 1:**
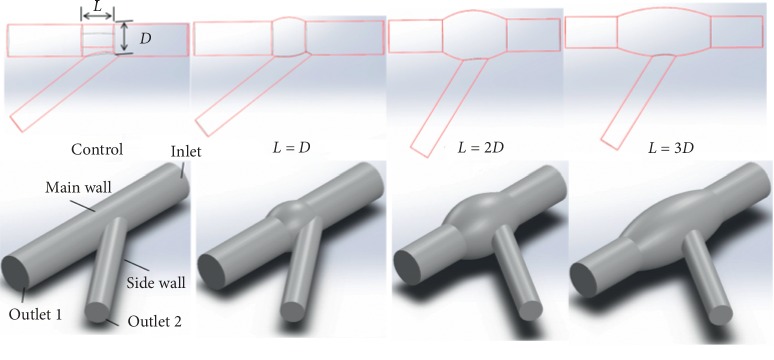
Schematic diagram of a vessel with enlarged segment at branch after the intervention of vascular stent.

Construct a series of ideal vessel models in SolidWorks (Dassault Systemes S.A, USA) with different enlargement percentage at bifurcation at the rate of 0% to 30% with the interval of 5% and different length (*L*) of enlarged area at diameter (*D*) of 1 to 3 times of the initial value based on the clinical threshold of vascular stent design. Take the blood vessel with enlargement rate of 0% as static control in each group of different diameters. Constructed models with clarification of parts and corresponding parameters are shown in Figure 1.

To sum up, the detailed geometry parameters of stents are listed in (Tables 1 and 2). The average value above is derived from 1171 cases of clinical data of PCI treatment [21].

### 2.2. Meshing in ICEM CFD

**Figure 2:**
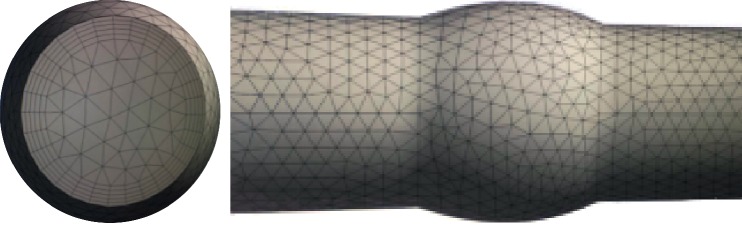
Mesh of “boundary layer” or prism layer drawn by Octree approach and mesh of enlarged vessel.

ICEM (ANSYS, Inc., Canonsbury, PA, USA) software was used to generate meshes of ideal models. Mesh is generated using the Octree approach to distinguish “boundary layer,” which is normally formed under nonnegligible adhesive force in blood flow. Global prism settings were assigned as: initial height 0, height growing ratio 1.1, and number of layers 5 [22]. Meshes were computed based on above settings. Mesh numbers are 413,578. The mesh of prism layer and the completed mesh of enlarged vessel model are shown in Figure 2.

### 2.3. Grid Independence Test

The enlarged vessel after the intervention of vascular stent with 5% enlargement at surface area and length of enlarged area at *L* = *D*, *L* = 2*D*, and *L* = 3*D* meshed by the control of relevance varies from 0, 10, to 100 is calculated in Ansys Fluent to determine the pressure in Group A (average pressure between inlet and outlet 2), B (average pressure between main wall and outlet 2), and C (average pressure between main wall and sidewall). The relative error based on the set of relevance equals to 100 is calculated to figure out whether the computing results is reliable and stable with the error beneath 1.00%. The results are listed in Table 3.

Most of the relative error based on the mesh control of relevance equals to 100 is beneath the limit value of 1.00%, which further implies the reliability of the computational results in our model. Thus, relevance equals to 100 is selected as the fixed index in following mesh control.

### 2.4. Simulation in Ansys Fluent

Numerical simulations were performed by employing ANSYS Fluent CFD (ANSYS Inc., Canonsburg, PA). Three-dimensional, laminar, steady-state, and pulsatile flows in all the modeled (Figure 2) coronary vessel geometries were analyzed, by solving incompressible continuity and Navier–Stokes equations specified in equations (2) and (3):

(2) $$\begin{array}{c} \rho \left(\frac{\partial \overset{⟶}{v}}{\partial t}+\left(\overset{⟶}{v}\cdot \nabla \right)\overset{⟶}{v}\right)=-\nabla P+\nabla \tau , \end{array}$$

(3) $$\begin{array}{c} \nabla \overset{⟶}{v}=0, \end{array}$$

where $\overset{⟶}{v}$ denotes the fluid velocity vector and *P* refers to the pressure. *ρ* denotes the blood density, which equals to 1050 kg/m,^3^ and *τ* denotes the stress tensor.

Set the inlet condition at the velocity of 0.36 m/s and outlet condition as “outflow,” which assumes zero pressure at the outlets with 61.3% flow stream from the outlet 1 and 38.7% flow from outlet 2 based on clinical data of coronary blood flow. A pressure-based solver was incorporated with a second-order upwind computing method for momentum spatial discretization.

The residual continuity was assigned as value of 1.0 × 10^−5^. Computational results of the hemodynamics parameter within initial vessel and each 6 vessels with the enlarged area length of *L* = *D* to *L* = 3*D* at different enlargement percentage of surface area varies from 5% to 30% at branches (in total as 19 enlarged vessels) were obtained through simulation.

### 2.5. Postprocessing in Ansys Fluent

Firstly, to guarantee the accuracy and reliability of the computed results, the Grid Independence Test (GIT) is done to calculate the pressure within same enlarged vessel at same location at different relevance value (respectively, are 0, 10, and 100). Relative error is calculated to examine if the computing process is independent of the meshing set, and 1.00% is chosen as the maximum limit of testifying standards towards relative error.

Secondly, due to the decrease in blood velocity, the WSS may fall below the alarming threshold at 0.5 Pa [23] that would cause atherosclerosis. Thus, the pressure at different parts (inlet, main wall, sidewall, and outlet 2) of the enlarged vessel is calculated to identify the improvement of blood supply, and the isosurface of pressure at 120 Pa is drawn to observe the existence of possible pressure concentration that may cause functional lapse of stent. Besides, WSS at different parts (main wall and sidewall) of the enlarged vessel is computed to determine the declining degree, and the isosurface of WSS at 0.5 Pa is drawn to figure out the potential risk for long-term atherosclerosis after implantation.

Thirdly, the average pressure between inlet and outlet 2 (recorded as A), the average pressure between main wall and outlet 2 (recorded as B), and the average pressure between main wall and sidewall (recorded as C) are computed and drawn in histogram to determine the superior design of stent enlargement parameters, which guarantees the maximum blood supply. The relevance of pressure fluctuation between Group A, Group B, and Group C is verified by *T* test.

In addition, the average WSS at main wall (recorded as E) and sidewall (recorded as F) is displayed in histogram to determine the superior design of enlargement parameters of stent with relatively higher value of WSS, which minimizes the danger of future atherosclerosis formation. The relevance of wall shear stress fluctuation between Groups E and F is verified by *T* test.

Finally, the optimized stent design with enlarged segment at branches to treat coronary bifurcation lesion is determined under the criteria of maximum blood pressure and WSS, minimum area of pressure concentration, and revealing limit of WSS under 0.5 Pa.

## 3. Results

### 3.1. Pressure

The average pressure between inlet and outlet 2 (A), main wall and outlet 2 (B), and main wall and sidewall (C) are listed in Table 4.

**Figure 3:**
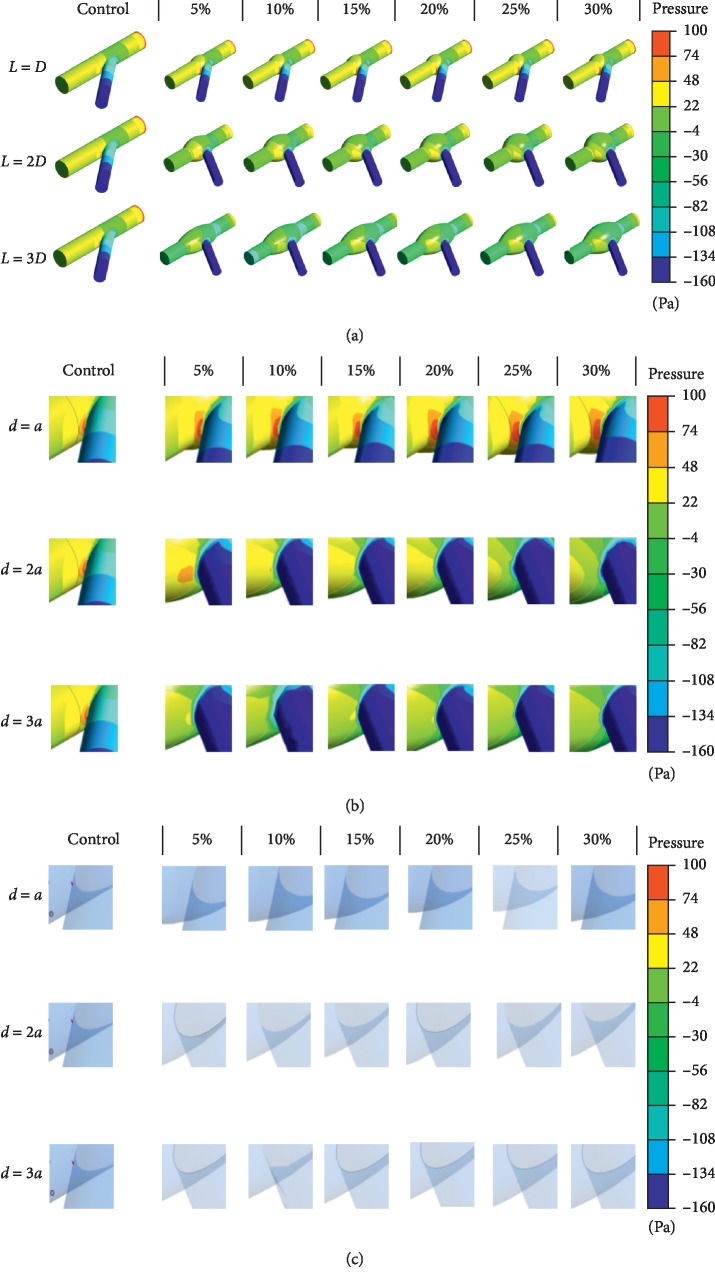
Pressure distribution. (a) Pressure distribution on the entire vessel. (b) Larger image of pressure distribution at the side branches and main walls. (c) Larger image of pressure distribution at the isosurface of pressure equals to 120 Pa.

The distribution of pressure at side branches and main walls as well as the isosurface of pressure equals to 120 Pa is shown in Figure 3. It can be preliminarily inferred that the pressure at side branches and pressure concentration area follows the pattern that decreasing with the rising of proportion of enlarged area and the length of enlarged vessels due to lower flow speed and higher blood pressure.

**Figure 4:**
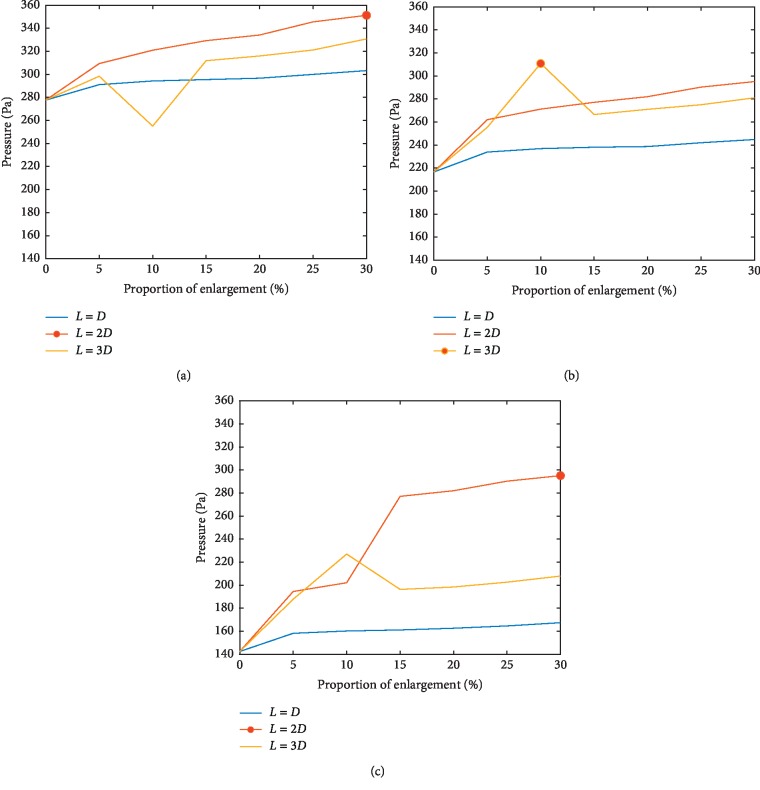
Pressure distribution (A, B, and C each shows pressure of different locations after implantation of enlarged vessels with different length of enlarged area varies from *L* = *D* to *L* = 3*D*, the maximum pressure value within each group is marked by red circle). (a) Pressure (A). (b) Pressure (B). (c) Pressure (C).

In order to quantitatively analyze the enhancement of blood pressure supply at branches, the histogram of pressure in Group A, B, and C at different proportion of enlarged area at branches with length of the enlarged area varies from *L* = *D* to *L* = 3*D* is shown in Figure 4.

It can be roughly inferred that with the increasing of proportion of enlarged segment area and enlargement length, the average pressure between inlet and outlet 2, inlet and main wall, and main wall and sidewall correspondingly enhanced, which suggests the improvement of blood supply to downstream organs. The maximum growth ratio appears in the stent design with parameter of 10% enlargement of surface area and *L* = 3D. Meanwhile, the enlarged design at branches reduces the pressure concentration rate, which may contribute to the minimal danger of stent instability due to the exceeding of stent strength limit during balloon dilation.

The results of *t*-test (*α* = 0.01) between pressure in Groups A, B, and C is shown in Table 5, which suggest that the fluctuating pattern of pressure between inlet and outlet 2, inlet and main wall, and main wall and sidewall has no significant statistical difference.

### 3.2. Wall Shear Stress

The average WSS at main wall (E) and sidewall (F) is listed in Table 6.

**Figure 5:**
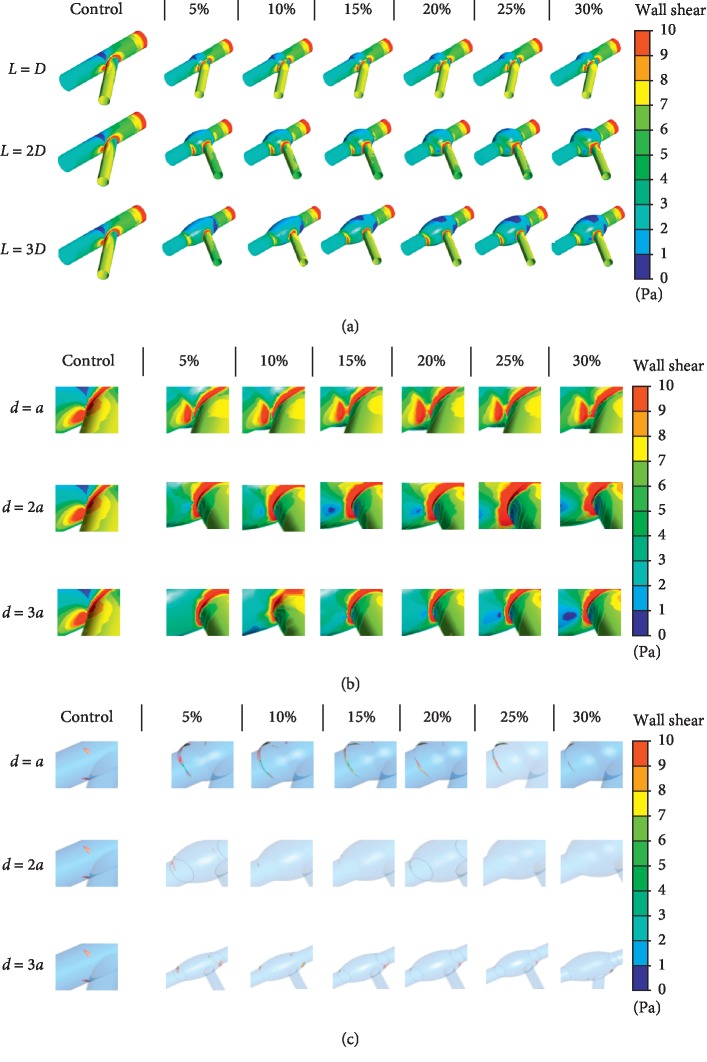
Wall shear stress distribution. (a) Wall shear stress distribution on the entire vessel. (b) Wall shear stress distribution at side branches and main walls. (c) Wall shear stress distribution on the side branches and main walls on isosurfaces at wall shear stress equals to 0.5 Pa.

The distribution of WSS at sidewall and main wall and the isosurface of WSS equals to 0.5 Pa is shown in Figure 5. It can be preliminarily inferred that the WSS at side branches decreases while the area of alarming threshold (WSS lower than 0.5 Pa, which may cause atherosclerosis) increases inevitably with the rising in proportion of enlarged area and the length of enlarged vessels due to higher blood pressure and lower flow speed (lower wall shear stress) as implied by Bernoulli's equation.

**Figure 6:**
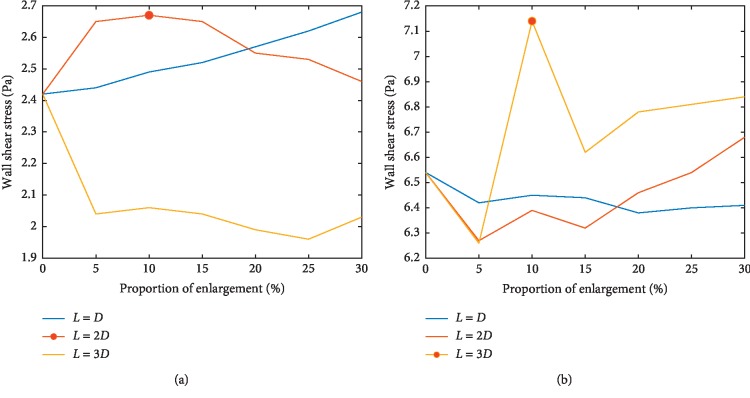
Wall shear stress distribution (E, F each shows wall shear stress at different locations of enlarged vessels with different length of enlarged area varies from *L* = *D* to *L* = 3*D*, the maximum wall shear stress value at main wall and sidewall is marked by red circle). (a) Wall shear stress (E). (b) Wall shear stress (F).

In order to quantitatively analyze the potential danger of atherosclerosis and plaque formation under the impact of decreasing WSS, the histogram of pressure in Groups E and F at different proportion of enlarged area at branches with length of the enlarged area varies from *L* = *D* to *L* = 3*D* is shown in Figure 6.

It can be roughly inferred that with the increasing of proportion of enlarged segment and length of enlarged main walls, the averaged WSS slightly goes down due to the declination of velocity and the area of WSS below alarming threshold, which may cause increasing danger of atherosclerosis is within controllable and reasonable levels. However, in some stent design, the value of WSS slightly goes up compared to the control group, which reduces the risk of embolism and compensates for the possible drawbacks of this design. The maximum growth ratio appears in the stent design with parameters at 30% enlargement of surface area and *L* = *D* at sidewall and 10% enlargement of surface area and *L* = 3*D* at main wall. Meanwhile, the enlarged design at branches inevitably increases the area of lower wall shear stress at the enlarged part but the total area of lower wall shear stress remains the same, which guarantees the stable control of potential risk of atherosclerosis.

The results of *t*-test (*α* = 0.01) between pressure in Groups E and F are shown in Table 7, which suggest that the fluctuating pattern of wall shear stress between main wall and sidewall has no significant statistical difference.

## 4. Discussion

The stenting at branch arteries is currently a difficult clinical problem to handle. To solve this problem, we proposed a novel stent design with enlarged segment at side branches and incorporated hemodynamics study to evaluate the hypothetical design. The results showed that the stent with enlarged segment improves blood supply pressure at side branches and enhances WSS at side branches. Meanwhile, the artery after implantation is in the absence of noticeable decrease in WSS at enlarged segment, and the pressure concentration problems at side branches are reduced due to the “Cushion” formed by the curvature segments. As a result, the hypothetical design guarantees adequate blood supply to side branches and minimal possibilities of atherosclerosis formation or blood blockage, ensuring the intervention stability during dilation with decreased pressure concentration.

The theoretical basis of the stent design in the present study is Bernoulli's equation, which is the fundamental energy conservation equation that applied to various research fields [24]. For instance, the wings of planes are designed as streamline structure to form levitated pressure with velocity on the bottom lower than the top [24, 25]. In the present study, we applied the energy conservation law to alter the partial segment shape of stent in order to enhance the perfusion pressure to side branches, which eliminates the adverse effects on physiological functions of downstream organs. The research results proved the accuracy and practical value of the hypothesis and are of significance to future stent design and intervention treatments of coronary branch lesions. The varying flow resistance at distal coronary artery can be taken into consideration in the future research and simulation to achieve more accurate estimation of pressure increase among specific patients.

To sum up, the lifted pressure at side branches, decreased distribution area of pressure concentration, and decreased wall shear stress within controllable level have rewarding clinical significance. Adequate blood supply at side branches is guaranteed, and stability of stent intervention is improved due to the pressure changes. Meanwhile, minimum decrease of wall shear stress on main enlarged walls and lifted wall shear stress after stent implantation may reduce the risk of atherosclerosis formation and restenosis rate.

Prior to future clinical applications, the hypothetical design can be further improved in materials and structural design like geometry shape of connecting ribs to obtain higher successful implantation rate, guaranteeing stent stability after balloon dilation, and minimizing the potential long-term risk caused by plaque shift and carinal shift.

### 4.1. Limitations

As a preliminary study, we adopted idealized models to investigate the practical value of using stent with enlarged segment to treat coronary branch lesions while the vessel structure in vivo is of irregularity and individual difference. Meanwhile, the set of rigid vessel wall condition in simulation is different from the *in vivo* situation. But as a preliminary study, we are aiming at eliminating the impact brought by the individual difference to validate the accuracy of our hypotheses at universal level. Future studies will be focusing on designing personalized stent structure based on different vessel locations, different anatomical structures of arteries, and different narrowed type for branch lesions.

## 5. Conclusion

The results have practical value for solving the shortcomings of existing single-stent or double-stent intervention strategy. Firstly, the enhanced single-stent technique can significantly reduce the implantation difficulty of double-stent technique, ensuring that the stents fit snugly against the vessel wall without changing the initial shape of the branched vessel as well as reducing the risk of reformation of the plaque. Meanwhile, the enlarged design at side branches based on the principle of Bernoulli's equation can theoretically improve the blood flow pressure by reducing the blood flow velocity to increase inadequate blood supply at the bifurcation artery, preventing the sharp decrease in wall shear stress, and contributing to the decrease in pressure distribution, which guarantees the stable implantation of stents and possibly leads to the reduction in restenosis rate.

## Figures and Tables

**Table 1:** Geometry parameters of enlarged vessels.

Main vessel	*D* (mm) (from 5% to 30%)	*L* (mm)
Control	3	3
*L* = *D*	3.78/3.84/3.90/3.98/4.04/4.10	3
*L* = 2*D*	3.78/3.84/3.90/3.98/4.04/4.10	6
*L* = 3*D*	3.78/3.84/3.90/3.98/4.04/4.10	9

**Table 2:** Geometry parameters of side branches.

Side branch	Diameter (mm)	Length (mm)	Angle (°)
	1.9	10	52

**Table 3:** Results of Grid Independence Test (GIT).

*L*	Relevance	A (Pa)	Error	B (Pa)	Error	C (Pa)	Error
*L* = *D*	0	286.50	1.56%	233.86	2.29%	158.16	0.15%
	10	289.68	0.47%	228.49	1.55%	158.39	0.11%
	100	291.05	NaN	230.22	NaN	158.32	NaN

*L* = 2*D*	0	308.01	0.44%	261.99	0.15%	193.58	0.47%
	10	308.01	0.44%	262.38	0.15%	193.58	0.47%
	100	309.38	NaN	262.38	NaN	194.49	NaN

*L* = 3*D*	0	298.40	0.22%	254.24	0.42%	187.54	0.15%
	10	297.73	0.54%	255.03	0.11%	186.83	0.53%
	100	296.79	NaN	255.32	NaN	187.82	NaN

**Table 4:** The average pressure at different locations (Groups A, B, and C).

	*L* = *D*			*L* = 2*D*			*L* = 3*D*		
	A (Pa)	B (Pa)	C (Pa)	A (Pa)	B (Pa)	C (Pa)	A (Pa)	B (Pa)	C (Pa)
Control	277.7	216.6	142.4	277.7	216.6	142.4	277.7	216.6	142.4
5%	291.1	233.9	158.2	309.4	262.0	194.5	298.4	255.3	187.8
10%	294.3	236.9	160.2	321.0	271.2	202.1	255.1	310.8	227.0
15%	295.5	238.1	161.1	329.3	277.1	277.1	311.9	266.5	196.3
20%	296.7	238.7	162.6	334.2	282.0	282.0	316.0	271.0	198.4
25%	300.0	242.0	164.6	345.6	290.3	290.3	321.2	275.0	202.6
30%	303.3	244.8	167.4	351.3	295.1	295.1	330.8	281.0	207.9

**Table 5:** Individual *T* test results between Groups A, B, and C (*H* = 1 suggests the close interrelationship within pressure fluctuating pattern).

	*L* = *D*			*L* = 2*D*			*L* = 3*D*		
	AB	BC	CA	AB	BC	CA	AB	BC	CA
H	1	1	1	1	1	1	1	1	1
P	3.09*e* − 08	1.32*e* − 09	8.58*e* − 13	2.10*e* − 03	3.73*e* − 04	1.33*e* − 06	5.50*e* − 03	2.94*e* − 04	1.12*e* – 06

**Table 6:** Raw data of average wall shear stress at different locations (Groups E and F).

	*L* = *D*		*L* = 2*D*		*L* = 3*D*	
	E (Pa)	F (Pa)	E (Pa)	F (Pa)	E (Pa)	F (Pa)
Control	2.42	6.54	2.42	6.54	2.42	6.54
5%	2.44	6.42	2.65	6.27	2.04	6.26
10%	2.49	6.45	2.67	6.39	2.06	7.14
15%	2.52	6.44	2.65	6.32	2.04	6.62
20%	2.57	6.38	2.55	6.46	1.99	6.78
25%	2.62	6.40	2.53	6.54	1.96	6.81
30%	2.68	6.41	2.46	6.68	2.03	6.84

**Table 7:** Individual *T* test results between Groups E and F (*H* = 1 suggests the close interrelationship within wall shear stress fluctuating pattern).

	*L* = *D*	*L* = 2*D*	*L* = 3*D*
	EF	EF	EF
H	1	1	1
P	1.19*e* − 18	3.42*e* − 16	5.58*e* − 14

## Data Availability

The hemodynamic simulation data used to support the findings of this study are included within the article.
